# Removal of Rhodamine B from Water Using a Solvent Impregnated Polymeric Dowex 5WX8 Resin: Statistical Optimization and Batch Adsorption Studies

**DOI:** 10.3390/polym12020500

**Published:** 2020-02-24

**Authors:** Moonis Ali Khan, Momina, Masoom Raza Siddiqui, Marta Otero, Shareefa Ahmed Alshareef, Mohd Rafatullah

**Affiliations:** 1 Chemistry Department, College of Science, King Saud University, Riyadh 11451, Saudi Arabia; mrsiddiqui@ksu.edu.sa (M.R.S.); 438203872@student.ksu.edu.sa (S.A.A.); 2 School of Chemical Engineering, Universiti Sains Malaysia, Engineering Campus, Nibong Tebal 14300, Penang, Malaysia; momina.anees@gmail.com; 3 CESAM - Centre for Environmental and Marine Studies, Department of Environment and Planning, University of Aveiro, Campus de Santiago, Aveiro 3810-193, Portugal; marta.otero@ua.pt; 4 School of Industrial Technology, Universiti Sains Malaysia, Main campus 11800, Penang, Malaysia

**Keywords:** modified polymeric resin, t-butyl phosphate impregnation, polymer based adsorbents, dye adsorption, response surface methodology

## Abstract

Herein, commercially available Dowex 5WX8, a cation exchange polymeric resin, was modified through solvent impregnation with t-butyl phosphate (TBP) to produce a solvent impregnated resin (SIR), which was tested for the removal of rhodamine B (RhB) from water in batch adsorption experiments. The effect of SIR dosage, contact time, and pH on RhB adsorption was studied and optimized by response surface methodology (RSM), interaction, Pareto, and surface plots. Scanning electron microscopy (SEM) and Fourier transform infrared spectroscopy (FTIR) were respectively used for characterizing SIR surface morphology and identifying active binding sites before and after RhB adsorption. SEM showed that the pristine SIR surface was covered with irregular size and shape spots with some pores, while RhB saturated SIR surface was non-porous. FTIR revealed the involvement of electrostatic and π–π interactions during RhB adsorption on SIR. Dosage of SIR, contact time, and their interaction significantly affected RhB adsorption on SIR, while pH and its interaction with dosage and contact time did not. The optimum identified experimental conditions were 0.16 g of SIR dose and 27.66 min of contact time, which allowed for 98.45% color removal. Moreover, RhB adsorption equilibrium results fitted the Langmuir isotherm with a maximum monolayer capacity (*q*_max_) of 43.47 mg/g.

## 1. Introduction

Textile industries are among the largest consumers of water, dyes, and different types of chemicals, resulting in the generation of large volumes of highly toxic effluents. The discharge of these effluents without prior treatment can be lethal to the environment. Usually, the textile effluents are rich in color, pH, chemical oxygen demand, inorganic salts, turbidity, and temperature [1]. According to the United States Environmental Protection Agency (USEPA), textile waste is mainly divided into four principal classifications, namely hard-to-treat, high volume, dispersible, and hazardous and toxic wastes [2]. Rhodamine B (RhB) is one of the most widely used cationic water-soluble organic dyes, and it is toxic to aquatic environments. It reduces sunlight penetration into water bodies, which can be lethal for aquatic life due to limited availability of oxygen for respiration [3,4,5]. Therefore, before effluent discharge, it is necessary to apply intensive treatment processes to minimize its concentration in water bodies. 

Membrane filtration, flocculation, biological treatments, photocatalytic oxidation, and adsorption [6,7,8,9,10] are some of the commonly used textile effluent treatment processes. On the other hand, the application of polymer materials in water treatment and selective sequestration has impressively developed in the last decades, with the production of novel materials and composites, post-polymerization modifications, introduction of functional groups, and development of supramolecular assemblies and nanomaterials [11,12,13,14,15,16]. However, these methods have their own limitations and efficiencies in terms of cost effectiveness. Recently, functionalized polymeric resins have become an alternative to commercial activated carbon and other adsorbents due to economic concerns and regeneration properties. Polymeric resins are characterized by their high surface area, moderate swelling, and narrow pore size distribution. In order to improve the adsorption characteristics of such resins, their surface properties can be modified using the advantage of adsorbate and adsorbent interaction. Mostly, ion-exchange resins are prepared by styrene divinylobenzene cross-linked co-polymer, which is comparatively lower in cost than activated carbon and serve several advantages as a matrix [17]. Additionally, the polystyrene based matrix has the potential to provide excellent chemical and physical stability together with resistance to degradation by oxidation or hydrolysis. Apart from ion-exchange resins, solvent extraction and liquid–liquid extraction, which work on the principle that solute distributes itself in a certain ratio with immiscible solvents, have attracted considerable attention in recent years [18]. The merits of solvent extraction include rapid and very selective separations that are usually highly efficient [18]. Solvent impregnated resins (SIRs) pose synergic merits of both ion-exchange and solvent extraction. A SIR is described as a liquid complexing agent dispersed homogeneously in a solid polymeric medium. In SIR removal processes, a specific solute is extracted from the aqueous phase to the organic phase inside the pores of the resin. The resin acts as carrier of the solvent and reduces the entrainment and irreversible emulsification that occur during solvent extraction [19]. Previously, a non-functional macroporous polymeric resin was used as a polymeric support for the removal of dyes from wastewater [20]. However, macroporous resins have lower retention capacity and slower kinetic diffusion compared to gel-type resins. Therefore, gel-type resins have higher removal efficiency than conventional macroporous polymeric resins. Moreover, the optimization of operational conditions for dye adsorption using SIR can improve the removal efficiency of dye. Thus, in this study, a gel-type Dowex cation exchange resin was used to remove RhB dye from aqueous solution. Moreover, the effect of interaction of operational conditions on the removal of RhB dye was also studied. Response surface methodology (RSM) is effective, reliable, and very comprehensive as compared to other conventional optimization processes [21]. It is a statistical tool that is very effective for designing, analyzing, and optimizing the effect of independent factors for the prediction of response output [21]. Therefore, the aim of this work was to investigate the operating conditions for removal of RhB dye using a 2^3^ full factorial design. Moreover, an equilibrium batch study was performed at optimum conditions to study the mechanism of dye removal using SIR.

## 2. Experimental

### 2.1. Chemicals, Reagents, and Adsorbent

Dowex 5WX8 gel-type cation exchange resin (BDH, England, UK) with particle size 0.39–1.00 mm and 50%–58% moisture content was used as an adsorbent. The resin contained a styrene divinyl benzene matrix, having sulfonic acid as a matrix active functional group. Rhodamine B (RhB: C_28_H_31_ClN_2_O_3_) (Sigma-Aldrich, Darmstadt, Germany) with respective color index and molecular weight 45170 and 479.2 g/mol, synonymously known as basic violet 10, was used as an adsorbate. Tributyl phosphate (TBP: C_12_H_27_O_4_P) was obtained from Sigma-Aldrich, Darmstadt, Germany. All the chemicals and reagents used during the study were of analytical reagent (A.R) grade or as itemized. Ultra-pure deionized (D.I: Millipore, Burlington, MA, USA) water was used throughout the study.

### 2.2. Synthesis of Solvent Impregnated Dowex 5WX8 Resin 

Initially, Dowex 5WX8 resin was washed with D.I water in order to remove inorganic impurities and monomeric material. Thereafter, the resin was overnight dried in an oven at 70 °C. The resin was impregnated with TBP (hydrophobic in nature) through the wet impregnation method reported elsewhere [22]. Briefly, undiluted TBP and resin in a volume to weight ratio of 6.0 was used to impregnate resin in a conical flask. Resin was aged for 24 h in TBP to achieve highest impregnation efficiency [20]. Further, the impregnated resin was separated from TBP through filtration and thoroughly rinsed with D.I water to remove unimpregnated traces of TBP. Then, solvent impregnated Dowex 5WX8 resin (SIR) was ready to use for adsorption studies. 

### 2.3. Characterization of Solvent Impregnated Dowex 5WX8 Resin 

Fourier transform infrared (FT-IR: Is10 Nicolet Thermo Scientific, Waltham, MA, USA) analysis was carried out to determine the available functional groups on Dowex 5WX8 resin and SIR (both pristine and RhB saturated) surfaces. The surface morphology of Dowex 5WX8 resin and SIR (both pristine and RhB saturated) was analyzed by scanning electron microscopy (SEM: Zeiss, model EVO Ma10, Oberkochen, Germany). 

### 2.4. Batch Scale Adsorption

The RhB adsorption studies over SIR were performed at room temperature by varying operation parameters viz. initial pH (pH_i_: 2–8), SIR dose (*m*: 0.1–0.5 g), and contact time (*t*: 5–30 min). A series of 10 mL RhB solutions of initial concentration *C_o_*: 100 mg/L were equilibration with 0.1–0.5 g SIR in 25 mL conical flasks over a shaker at 230 rpm. At predetermined contact times, solid/solution phases were separated, and residual RhB concentrations in solutions were determined by using a Shimadzu UV-Visible Spectrophotometer at λ_max_: 554 nm. The amount of RhB adsorbed at any time *t* onto SIR was calculated as:

(1) $$Adsorbed\;concentration\;at\;time\;t\;\left(q_{t},mg/g\right)=\left(C_{o}-C_{t}\right)\times \frac{V}{m}$$

where V (L) is the volume of RhB solution, *C_o_* (mg/L) is the initial RhB concentration, *C_t_*(mg/L) is the remaining RhB concentration in solution at any time *t*, and *m* (g) is the mass of SIR. 

The amount of RhB adsorbed on SIR at equilibrium, which was attained in 30 min under shaking, was calculated as:

(2) $$Adsorbed\;concentration\;at\;equilibrium\;\left(q_{e},\;mg/g\right)=\left(C_{o}-C_{e}\right)\times \frac{V}{m}$$

where *C_e_* (mg/L) is the equilibrium concentration of RhB in solution.

The decolorization efficiency (D.E, %) was calculated as follows:

(3) $$Decolourization\;efficiency\;\left(D.E.\;\%\right)=\;\frac{C_{o}-C_{e}}{C_{o}}\times 100\;$$

### 2.5. Design of Experiments and Optimization of Parameters

Two level (low level –1 and high level +1) factorial design (2^3^) of response surface methodology (RSM) was applied for three independent variables (factors), namely the operational parameters dosage of SIR (A), contact time (B), and initial pH (C), to predict D.E. (%) (response factor, *y*) from RhB dye solution using solvent impregnated resin (Table 1). A total 12 runs including 4 times of replication for center point was carried out (2^3^ = 8 runs; 8 runs + 4 replications for center point = 12 runs) using Design Expert (6.0.10) (Stat Ease, Minneapolis, MN, USA). Suitable approximation can be determined for the true functional relationship between the process response, *y*, and the set of factors by first order model or second order model. When the response linearly varies with the independent variable, then the first order model, which is given by Equation (4), is satisfied.

(4) $$y=\;\beta_{0}+\beta_{1}x_{1}+\ldots \beta_{k}x_{k}+\varepsilon$$

where, *y* is the response, $\beta_{0}$ is the offset term, $\beta_{1}$, …, $\beta_{k}$ are the effect term, *x_1_, …, ** x_k_* are the independent variables, and ε is the random error term. When a curvature is detected in the system, second order model is selected and expressed by the following equation (Equation (5)):

(5) $$y=\;\beta_{0}+\overset{k}{\underset{i=1}{\sum }}\beta_{1}x_{1}+\overset{k}{\underset{i=1}{\sum }}\beta_{ii}x_{i}^{2}+\overset{n}{\underset{i=1}{\sum }}\overset{n}{\underset{i<j}{\sum }}\beta_{ij}x_{i}x_{j}\;$$

where *y* is the predicted response, $\beta_{0}$ is the constant, $\beta_{1}$ is the linear effect, $\beta_{ii}$ is the square effect, and $\beta_{ij}$ is the interaction effect.

For the optimization of experimental design, the statistical software Minitab 16 was used. The results were analyzed by estimating the response of the dependent response variable to obtain the effects, coefficient, and other statistical parameters. The conditions for optimization of adsorption process were obtained from Minitab 16 (Minitat LLC., Penn State University, PA, USA) as well. By using the analysis of variance (ANOVA), the determination coefficient (r^2^) and statistical significance were determined.

### 2.6. Isotherm Modeling

Langmuir and Freundlich isotherm models in linearized forms were fitted to data on RhB adsorption onto SIR. The Langmuir isotherm model, which assumes monolayer adsorption over homogenous sites on the adsorbent surface and equal activation energy for each molecule, is given by Equation (6) in its linearized form:

(6) $$\frac{1}{q_{e}}=\frac{1}{q_{max}K_{L}C_{e}}+\frac{1}{q_{max}}$$

where *q_e_* (mg/g) is the amount of RhB adsorbed on SIR, *C_e_* (mg/L) is the saturated amount of RhB adsorption at equilibrium concentration, and *q_max_* (mg/g) is the maximum monolayer adsorption capacity of RhB on SIR. The constants *K_L_* and *q_max_* can be calculated from a linear plot of 1/*q_e_* vs. 1/*C_e_*. The characteristics of the fitting to the Langmuir equation are given by a dimensionless number, *R_L_* (Equation (7)), which indicates the type of isotherm to be irreversible (*R_L_* = 0), favorable (0 < *R_L_* < 1), linear (*R_L_* = 1), or unfavorable (*R_L_* > 1) [23].

(7) $$R_{L}=\frac{1}{1+K_{L}C_{o}}$$

The Freundlich isotherm, which is an empirical model, is usually associated with multilayer adsorption of RhB molecules over heterogenous adsorption sites and can be expressed in linearized form as:

(8) $$\log q_{e}=\log K_{F}+\frac{1}{n}\;\log\;C_{e}$$

where *K_F_* is a Freundlich constant, and *n* is a parameter related to the binding strength changes with the adsorption density. If 1/*n* = 0, it indicates that the extent of adsorption is independent between two phase concentration; 1/*n* < 1 indicates favorable chemical adsorption; 1/*n* > 1 indicates a cooperative adsorption [23].

## 3. Results and Discussion

### 3.1. Pre and Post-Adsorption Characterization

**Figure 1:**
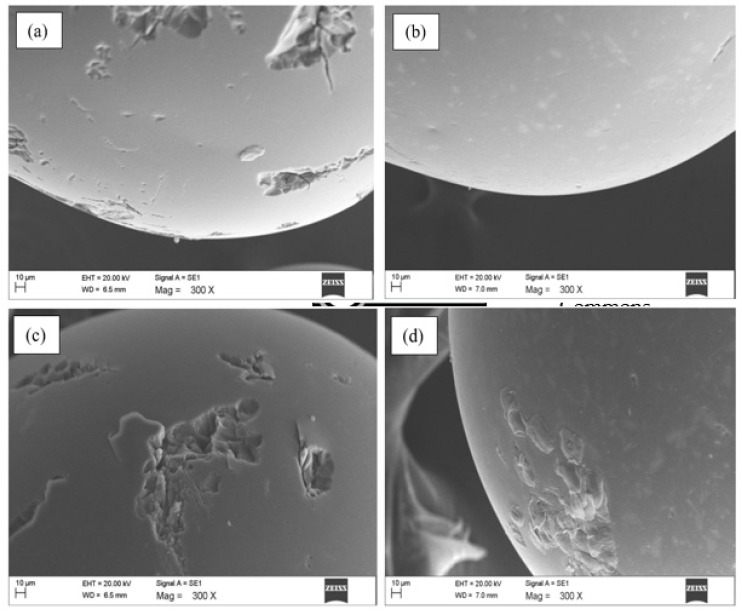

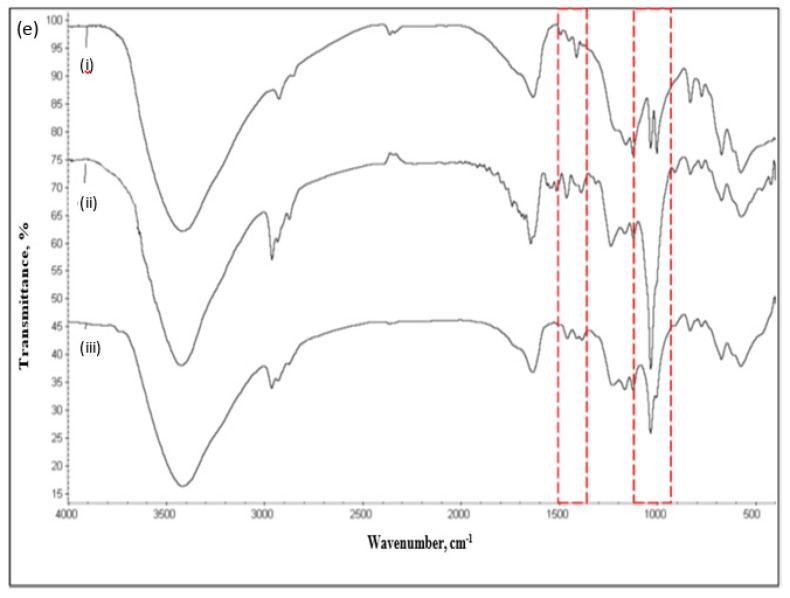
Scanning electron microscopic (SEM) images of raw Dowex 5WX8 polymeric resin (**a**), pristine SIR (**b, c**), RhB saturated SIR (**d**), and Fourier transform infrared (FT-IR) spectra of raw Dowex 5WX8 polymeric resin (**i**), pristine SIR (**ii**), and RhB saturated SIR (**iii**) (**e**)**.**

The surface morphologies of Dowex 5WX8 resin and SIR (both pristine and RhB saturated) were analyzed using SEM with 300X magnification, as illustrated in Figure 1a–d. The raw Dowex 5WX8 resin has a smooth surface with some pores (Figure 1a). After TBP solvent impregnation over raw Dowex 5WX8 resin, the whole SIR surface was covered with spots of irregular size and shape (Figure 1b). This confirms successful impregnation of raw Dowex 5WX8 resin [24]. The structural pores after impregnation remained unchanged, as shown in Figure 1c. These pores were well occupied by RhB molecules during adsorption, displayed by protruding occupation of pores (Figure 1d). The FT-IR spectrum of raw Dowex 5WX8 resin (Figure 1e) showed a strong band centered at 3420 cm^–1^, ascribed to hydroxyl (–OH) group stretching, due to the presence of internal moisture in raw Dowex 5WX8 resin. The conjoint bands at 2927 and 2852 cm^–1^ were associated with C–H stretching vibrations for saturated aliphatic species. The bands between 1483 and 1510 cm^–1^ were due to CH_3_ deformation in amino acid or hydrochloride compounds in raw Dowex 5WX8 resin. Moreover, the bands at 1010 and 1033 cm^–1^ represented C–O stretching of cyclic alcohol in raw resin. However, after impregnation of raw Dowex 5WX8 resin with TBP, the band at 1033 cm^–1^ became sharp and intense due to P–O–C stretching, thus confirming the attachment of phosphorous with C–O. A band at 1226–1237 cm^–1^ was due to P=O stretching in the phosphate group, and a band at 1383–1388 cm^–1^ showed CH_3_ deformation in the t-butyl group. The presence of P=O and P–O–C groups, and CH_3_ deformity indicate successful impregnation of raw Dowex 5WX8 resin to form SIR. After RhB adsorption on SIR, the band at 1033 cm^–1^ was displaced by a low intensity band at 1034 cm^–1^, confirming its involvement in binding dye molecules during adsorption. 

### 3.2. Screening of Process Independent Variables

In this batch adsorption study, the interaction of operational parameters for the removal of RhB using SIR was examined. A total of 12 experimental runs were optimized using three dominant parameters for the removal of RhB from aqueous solution, which was calculated using Equation (3). It was used to achieve improved adsorption capacity of SIR by possible interaction of operational parameters (Table S1, Supplementary Materials). The results showed that two of the considered operational parameters, specifically dosage of SIR and contact time, influenced the removal of RhB from aqueous solution. Additionally, the surface modification of Dowex 5WX8 resin by impregnation to SIR led to improved surface characteristics for the removal of RhB. 

The effect of variable interaction during the adsorption process was carried out using analysis of variance (ANOVA). Then, 2^2^ fractional factorial designs were used to study the selected factors. The purpose of carrying out the fractional factorial design was to determine the factors that had a significant effect on D.E. (%). ANOVA for the fractional factorial design is given in Table 2. The ANOVA and response surface regression of D.E. (%) is tabulated in Table 3. The effect of pH for aqueous phase was found to be insignificant due to the p-value of 0.314, which was greater than 0.05. Moreover, the negative coefficient (–1.08) of pH pointed towards a decrease in adsorption efficiency as the pH increased. The overall prediction of the output model in terms of operational parameters showed that the model was suitable for predicting the adsorption of RhB on SIR (p < 0.05). The respective p-values of SIR dosage and contact time were 0.063 (nearer to 0.05) and 0.012, which showed that both parameters significantly influenced D.E. (%). Furthermore, the linear effects of both SIR dosage and contact time indicated that they were suitable for improving adsorption efficiency. 

However, quadratic coefficients of SIR dosage and contact time inhibited the performance of RhB adsorption from aqueous solution (p > 0.05). Moreover, the negative coefficient of interaction of dosage and contact time was helpful in increasing adsorption efficiency. The polynomial first order and interactive regression model equation was developed using Minitab software. Therefore, the model equation is given as:

(9) y=91.374+11.215A+17.214B−20.055AB

where *y* is the D.E. (%) of RhB. In the aforementioned equation, a synergistic effect was indicated by the positive sign, while an antagonistic effect was indicated by the negative sign [25]. The ANOVA for the model is given in Table 4. It was deduced that the color removal was significant at 95% (*p* < 0.05) confidence level, which shows the validity of the model for RhB adsorption onto SIR.

**Figure 2:**
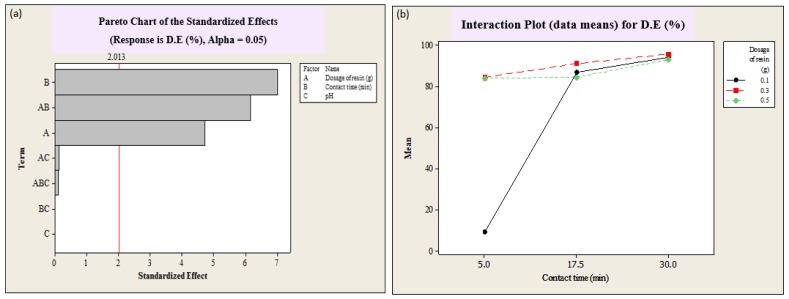
Pareto chart showing the effects and interactions of operational variables, namely dosage of resin (g), contact time (min) and pH, on the decolorization efficiency (D.E. (%)) by SIR (**a**)**,** and interaction plot of decolorization efficiency (D.E. (%)) by SIR versus contact time (min) for the different dosages of resin, namely 0.1, 0.2, and 0.3 g of SIR (**b**).

The determination of coefficient (r^2^) was used to evaluate the quality of the developed model [7,26]. The r^2^ value of the color removal was 82.17%, which means that 0.8217 of total variation was explained by the model, while 17.83% of the variation was left unexplained. The importance of the effects of the operation variables and their interaction can be best described by the Pareto chart, as shown in Figure 2a. A student’s t-test was performed to determine whether the calculated effects were significantly different from zero; these values for each effect are shown in the Pareto chart by horizontal columns [7]. The t-value for 95% confidence level was 2.013. The values exceeding the reference line were considered as significant for 95% confidence level, whereas values below the reference line were considered as insignificant. As shown in Figure 2a, the two parameters, dosage of SIR (A) and contact time (B), as well as their interaction (AB) were found to be significant at the 0.05 level. However, the effect of pH (C) and its interaction with dosage (AC) and contact time (BC) were below the reference line, which points to their insignificance for D.E. (%). According to previous studies, the effect of increasing pH was considered as favorable for the removal percentage of RhB [27,28]. However, in this study the effect of pH was not found to be significant. Therefore, the effects of dosage, contact time, and their interaction, which resulted in 97.45% of dye adsorption efficiency, were studied. 

**Figure 3:**
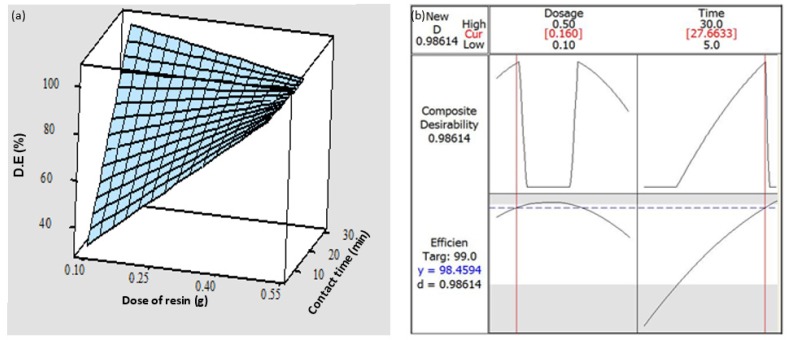
Surface plot showing the decolorization efficiency (D.E. (%)) as a function of the dosage of resin (g) and contact time (min) under a shaking speed of 230 rpm and pH 3.6 (**a**), and optimization plot for the determination of the optimum conditions, namely dosage of resin (g) and contact time (min), for a maximum decolorization efficiency (D.E. (%)) by SIR (**b**).

The interaction of contact time and dosage of resin were described by interaction plots, illustrated in Figure 2b. The interaction plots show the D.E. (%) versus the contact time (min) for each dosage of SIR. It was found that as the contact time increased, the D.E. (%) increased and reached its maximum for a 0.3 g dosage of SIR. The interaction between contact time and SIR dosage improved the adsorption efficiency of RhB, as shown in Figure 3a. The response surface plots show the estimated value of D.E. (%) (the height of the surface represents the value of D.E. (%)) as a function of the independent variable. It must be highlighted that the surface plots represent the same results as observed in interaction plots.

### 3.3. Optimization of Experiment

A standard RSM design called central composite design (CCD) was used to optimize the operating parameters. Optimum conditions of effective parameters with minimum number of experiments can be determined by this statistical technique. This method is also suitable to analyze the interaction and relationship between each parameter. The optimum conditions for the removal of RhB dye were obtained from the screening of operational parameters (Table S1, Supplementary Materials). The optimum operational parameters for D.E. (97.45%) were achieved with 0.3 g SIR dosage and 30 min contact time. Since contact time played a major role in the extraction process [20], therefore extraction efficiency of TBP impregnated SIR increased steadily with time until it reached equilibrium. Moreover, an increasing amount of SIR increases the D.E. (%) because low amounts of SIR contain low amounts of extractant [20]. However, high amounts of SIR and TBP cause an increase of the dye solution acidity. Therefore, concentrated acidic medium causes back-extraction of extractant–dye complex, thus resulting in higher dye concentration [29].

The optimization plot, which is displayed in Figure 3b, was used to find optimum conditions for RhB removal by SIR. From the analysis of experimental data obtained, the optimum identified conditions were 0.16 g of SIR dose and 27.66 min of contact time. With the application of such optimum conditions, the predicted value of D.E. was 98.45%, which was experimentally verified to be fulfilled with a deviation of ± 0.1%. 

### 3.4. Adsorption Isotherm

The adsorption isotherm is used to study the mechanism and pattern of adsorption at liquid-phase equilibrium [21,30,31]. Fittings of equilibrium data for the adsorption of RhB on SIR by Langmuir and Freundlich models were determined in this work. Linear plots of Langmuir and Freundlich isotherms and the respective parameters are shown in Figure S1 (Supplementary Materials). As can be seen, equilibrium results fitted the Langmuir isotherm (r^2^ = 0.99) but not the Freundlich model (r^2^ = 0.087). Therefore, it may be assumed that the adsorption of RhB on SIR was the monolayer on the surface of SIR, where the active sites and energies were homogenously distributed [32,33]. The fitted maximum monolayer adsorption capacity (*q_max_*) of RhB on SIR was found to be 43.47 mg/g, and *K_L_* was 0.0126 L/mg. Therefore, adsorption of RhB on SIR was found to be favorable because R_L_ was calculated (Equation (7)) to be 0.284, which is greater than 0 and smaller than 1.

### 3.5. Adsorption Mechanism

**Figure 4:**
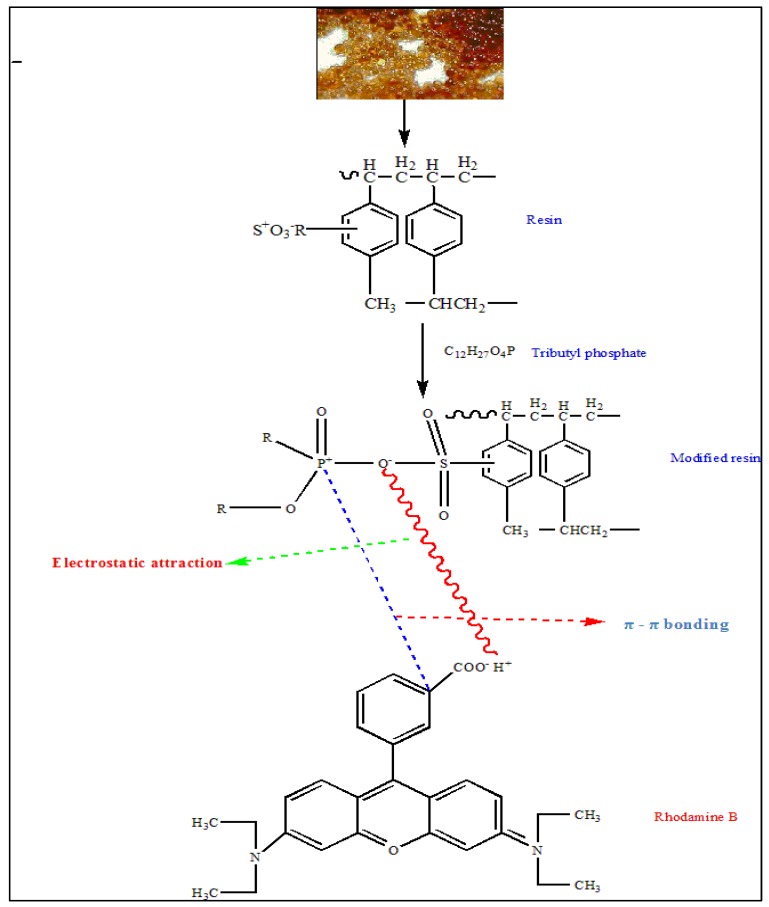
Schematic representation of SIR production and RhB adsorption mechanism on SIR.

Dowex 50WX8, a cation exchange polymeric resin, was used in this study. It contains cross-linked styrene divinyl benzene co-polymer with sodium sulfonate groups as ion-exchange sites. The resin was impregnated with TBP, which contains certain functional groups that have significant influence on the adsorption of RhB dye (as revealed by FT-IR results in Figure 1e). Therefore, an increased RhB adsorption can be achieved using the interaction between the adsorbate (RhB) and modified resin (SIR). The structure and functional groups present on modified SIR resin were the main factors responsible for the adsorption of RhB dye on this resin. Rhodamine B dye has amino and carboxylic functional groups, which can be involved on its adsorption on modified SIR. According to FTIR results, the peaks for P–O–C disappeared after adsorption of dye on impregnated resin and were replaced by the C–O group of the RhB dye. On the other hand, phosphate was not detected in solution after the adsorptive removal of RhB. Therefore, phosphate groups might be responsible for binding the positively charged dye ions by modified SIR resin. Furthermore, the possible interaction that might be occurring between modified SIR resin and RhB dye can be electrostatic and π–π bonding, as shown in Figure 4.

## 4. Conclusions

A TBP impregnated polymeric resin was produced in this work and tested for the removal of RhB from water. FT-IR analysis showed the presence of a phosphate functional group on the surface of the solvent impregnated resin (SIR), which was indicative of successful impregnation of TBP over the resin. The effect of operational conditions, namely pH, adsorbent dosage, and contact time, on the adsorption of RhB onto SIR was studied and optimized. It was found that pH does not have a significant effect on the D.E. (%). The maximum color removal obtained was 97.45% at 100 mg/L of initial dye concentration, 230 rpm of shaking speed, pH 3.6, 0.3 g of resin dosage, and 30 min of contact time. The optimum conditions for the adsorption of RhB by SIR were identified as 0.16 g of SIR resin and 27.66 min of contact time, which gave 98.45% of color removal. The adsorption data fitted well the Langmuir isotherm model, which pointed to monolayer adsorption on the SIR surface, with homogeneous distribution of active sites and energies. Furthermore, interaction of RhB and SIR was inferred to be electrostatic and π–π bonding.

## Figures and Tables

**Table 1:** Factors, levels, and ranges of the parameters considered for the factorial design.

Terms	Factors	Levels		
		–1	0	+1
A	SIR dose (g)	0.1	0.3	0.5
B	Contact time (min)	5	17.5	30
C	Initial pH of RhB solution	2	5	8

**Table 2:** ANOVA analysis for the fractional factorial design carried out to determine the factors that have significant effect on the decolorization efficiency (D.E. (%)).

Term	Coefficient	SE Coefficient	T-value	P-value
Constant	71.78	0.9575	74.97	0.000
A	18.90	0.9575	19.74	0.000
B	21.53	0.9575	22.48	0.000
C	–1.08	0.9575	–1.13	0.341
AB	–20.93	0.9575	–21.86	0.000
BC	0.07	0.9575	0.08	0.943
AC	0.24	0.9575	0.25	0.817
ABC	0.99	0.9575	1.03	0.378

SE Coefficient = standard error of the coefficient.

**Table 3:** Estimated regression coefficients and ANOVA for optimization of decolorization efficiency (D.E. (%)).

Term	Coefficient	SE Coefficient	T-value	P-value
Constant	91.374	5.156	17.722	0.000
A	11.215	5.069	2.212	0.063
B	17.214	5.069	3.396	0.012
A^2^	–12.117	7.472	–1.622	0.149
B^2^	–6.863	7.472	–0.918	0.389
AB	–20.055	6.209	–3.230	0.014

SE Coefficient = standard error of the coefficient.

**Table 4:** ANOVA analysis of the model for the removal of RhB using the produced SIR.

Source	Degree of Freedom	Sum of Squares	Mean Squares	F-value	P-value
Regression	5	4972.66	994.53	6.45	0.015
A	1	754.69	754.69	4.89	0.063
B	1	1777.96	1777.96	11.53	0.012
A^2^	1	701.09	405.48	2.63	0.149
B^2^	1	130.08	130.08	0.84	0.389
AB	1	1608.84	1608.84	10.43	0.014
Residual error	7	1079.36	154.19		
Lack of fit	3	1052.53	350.84	52.30	0.001
Pure error	4	26.83	6.71		
Total	12	6052.02			
r^2^ = 82.17% r^2^ (adjusted) = 69.43%					

## Supplementary Materials

The following are available online at https://www.mdpi.com/2073-4360/12/2/500/s1, Figure S1: Langmuir (a) and Freundlich (b) plots for the adsorption of RhB on SIR; Table S1: Results of response surface methodology design.
