# Supplementary material for: Removal of Rhodamine B from Water Using a Solvent Impregnated Polymeric Dowex 5WX8 Resin: Statistical Optimization and Batch Adsorption Studies
Source: Polymers (Basel). 2020 Feb 24;12(2):500. doi: 10.3390/polym12020500 (PMC7077689; doi:10.3390/polym12020500)
Supplement: Supplementary file 1 [file polymers-12-00500-s001.pdf]

Supplementary Data

Removal of Rhodamine B from water using a Solvent Impregnated Polymeric Dowex 5WX8 Resin: Statistical Optimization and Batch Adsorption Studies

Moonis Ali Khan [1,*], Momina [2], Masoom Raza Siddiqui [1], Marta Otero[3], Shareefa Ahmed Alshareef [1], Mohd Rafatullah [4,*]

[1] Chemistry Department, College of Science, King Saud University, Riyadh 11451, Saudi Arabia
[2] School of Chemical Engineering, Universiti Sains Malaysia, Engineering Campus, Nibong Tebal, 14300, Penang, Malaysia
[3] Department of Environment and Planning & CESAM, University of Aveiro, Campus de Santiago, Aveiro 3810-193, Portugal
[4] School of Industrial Technology, Universiti Sains Malaysia, Main campus, 11800, Penang, Malaysia

* Correspondence: mokhan@ksu.edu.sa, moonisalikhan@gmail.com (M.A.K.); mrafatullah@usm.my (M.R.)

Corresponding Author: Moonis Ali Khan
E-mail addresses: mokhan@ksu.edu.sa, moonisalikhan@gmail.com (M.A.Khan); mrafatullah@usm.my (M. Rafatullah)

Table 1. Results of response surface methodology design.

A<br>Dosageof Resin (g)	B<br>Contact Time (min)	C<br>Colour Removal (%)
0.1	5	9.09
0.5	5	83.98
0.1	30	94.41
0.5	30	89.08
0.1	17.5	86.90
0.5	17.5	84.63
0.3	5	84.59
0.3	30	97.45
0.3	17.5	85.19
0.3	17.5	91.36
0.3	17.5	86.96
0.3	17.5	90.41
0.3	17.5	89.94

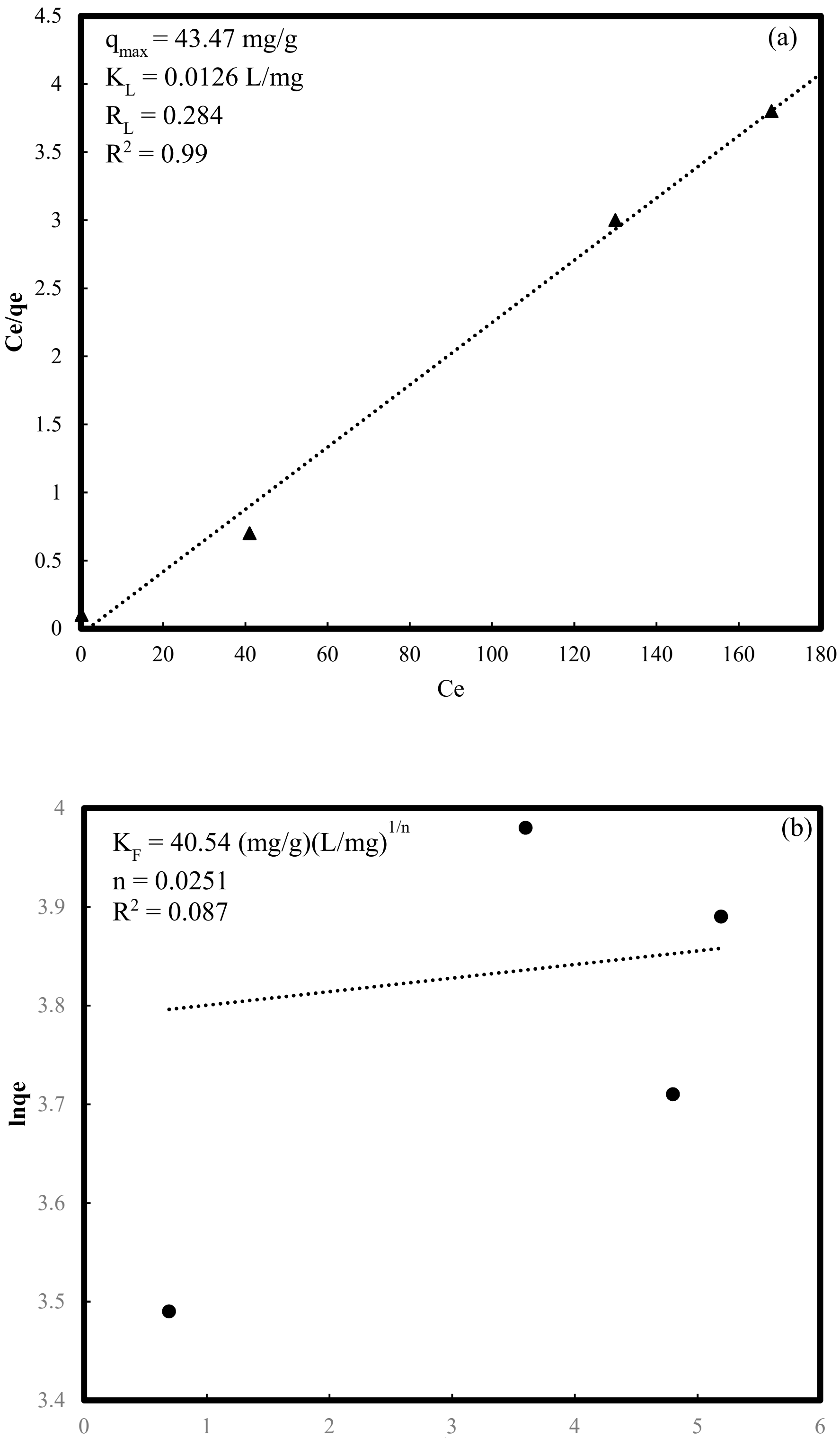

Figure S1. Langmuir (a) and Freundlich (b) plots for the adsorption of RhB on SIR.
